# Stepwise tapering of remifentanil at the end of surgery decreased postoperative pain and the need of rescue analgesics after thyroidectomy

**DOI:** 10.1186/s12871-015-0026-8

**Published:** 2015-04-08

**Authors:** Sun Sook Han, Sang Hwan Do, Tae Hee Kim, Won Joon Choi, Ji Sup Yun, Jung Hee Ryu

**Affiliations:** 1 Department of Anesthesiology & Pain Medicine, Kangbuk Samsung Hospital, Sungkyunkwan University School of Medicine, Seoul, South Korea; 2 Department of Anesthesiology & Pain Medicine, Seoul National University College of Medicine, Seoul, South Korea; 3 Department of Surgery, Kangbuk Samsung Hospital, Sungkyunkwan University School of Medicine, Seoul, South Korea; 4 Department of Anesthesiology & Pain Medicine, Seoul National University Bundang Hospital, Seongnam-si, Gyeonggi-do South Korea

**Keywords:** Anesthetic volatile-desflurane, Analgesics opioid- remifentanil, Complications-hyperalgesia

## Abstract

**Background:**

This study was designed to investigate whether stepwise tapering of remifentanil at the end of surgery could decrease postoperative pain scores and requirements of rescue analgesics after remifentanil-desflurane anesthesia in patients with thyroidectomy.

**Methods:**

Sixty two patients undergoing thyroidectomy under general anesthesia were randomly allocated into two groups. All patients were anesthetised with desflurane and high-dose remifentanil. Remifentnail was infused at the rate of 0.3 μg/kg/min until the end of surgery in patients of the control group (group A) whereas remifentanil was tapered gradually from 0.3 to 0.1 μg/kg/min until the end of surgery for at least 30 minutes in patients with group B. Pain scores (0–100 numerical rating scale, NRS), rescue analgesic requirements and adverse events were assessed at 30 min, 2 h, 6 h, 12 h, and 24 h after operation.

**Results:**

There was a significant decrease in pain scores at 30 min (20 [0–80] *vs*. 50 [0–100], P = 0.002) and 2 h (30 [10–60] *vs*. 40 [20–80], P = 0.018) after surgery in group B compared with group A. In addition, rescue analgesics are less required in group B than in group A postoperatively (2 [1-3] *vs*. 3 [2,3], P = 0.039). There were no significant differences in adverse events between the two groups.

**Conclusions:**

Tapering of remifentanil at the end of surgery decreased postoperative pain scores immediately after thyroidectomy with desflurane and high-dose remifentanil anesthesia.

**Trial registration:**

Clinical Research information Service (CRiS, registration number KCT0000589).

## Background

Opioids are commonly used supplements during general anesthesia to reduce requirement of anesthetics and to attenuate sympathetic response to noxious stimuli [1]. Among various opioids, remifentanil, a selective μ-opioid agonist, is widely administered due to its rapid onset, short acting period and predictable rapid recovery [1]. However, remifentanil infusion during anesthesia has been associated with increased early postoperative pain and rescue analgesics due to the development of opioid induced hyperalgesia and acute opioid tolearance [2,3]. This could be a challenge for postoperative pain control and thereby result in increase of postoperative analgesic requirements [2,4,5].

The mechanism underlying hyperalgesia after opioid infusion is still unclear. The animal experimental results showed that both peripheral and central changes in nociceptive processing are involved in this process [6]. Central sensitization through activation of the N-methyl-D-aspartate (NMDA) receptors is considered to be responsible for opioid induced hyperalgesia [7,8]. Excitatory amino acids such as glutamate, aspartate, and substance P are released from excitatory synapse membranes through central sensitization and then activate postsynaptic NMDA receptors [9]. The recent study showed that remifentanil induced rapid and persistent increases in NMDA responses at clinically relevant concentrations [10]. Another animal experiment also showed that abrupt withdrawal from μ opioid receptor induced long-term potentiation at the synapse of the pain pathways, which is related with opioid induced hyperalgesia [11].

Several pharmacologic methods including NMDA antagonists, nonsteroidal anti-inflammatory drugs (NSAIDs) and alpha agonists have been investigated to prevent opioid induced hyperalgesia. Besides pharmacologic preventions, the authors hypothesized that stepwise tapering of high-dose remifentanil at the end of surgery could prevent long-term potentiation at synapses of the pain pathways [11] and thus diminish postoperative pain and rescue analgesics during postoperative period. In this study, the effect of intraoperative tapering of remifentanil on postoperative pain and rescue analgesics was investigated in patients undergoing thyroidectomy with desflurane-remifentanil anesthesia.

## Methods

### Patients

The protocol of this prospective, randomised, and single-blind investigation was approved by the Institutional Review Board of Kangbuk Samsung Hospital (IRB No. KBC12145) and then registered with the Clinical Research information Service (CRiS, registration number KCT0000589). Informed consent was obtained from each patient. A total of 62 American Society of Anesthesiologist (ASA) physical status I-II patients, aged 19–70 years, and scheduled for thyroidectomy under general anesthesia from November 2012 to January 2013 were enrolled. Patients with hypersensitivity or allergy with opioids, alcohol or opioids abuse, chronic pain, or psychiatric disease were excluded from the study. Patients were instructed with the use of numerical rating scale (NRS, 0 = no pain to 100 = most severe pain) at the preoperative visit.

### Anesthesia

All patients were premedicated with i.v. 0.03 mg/kg midazolam and i.m. glycopyrrolate 0.2 mg 30 minutes before arriving at the operating room. In the operating room, standard monitors (electrocardiogram, noninvasive blood pressure, pulse oximetry and capnography) and spectral entropy for monitoring depth of anesthesia were applied. Anesthesia was induced with i.v. 5 mg/ kg thiopental, 0.3 μg/kg/min remifentanil and desflurane 6 vol% followed by 0.6 mg/kg rocuronium to facilitate endotracheal intubation. Maintenance of anesthesia consisted of desflurane 3.0-6.0% (end-tidal concentration) with medical air in oxygen (FiO_2_ = 0.5) and remifentanil to maintain spectral entropy value of 40–60. Patients were mechanically ventilated to maintain an end-tidal concentration of carbon dioxide between 35–40 mmHg throughout the surgery. Muscle relaxation was achieved with 0.15 mg/ kg rocuronium to maintain 1–2 twitches in response to train-of-four stimulation of the ulnar nerve. Esophageal temperature was monitored and maintained at 36-37 °C throughout the operation using a warming pad. At the end of the surgery, desflurane and remifentanil administration were discontinued and muscle relaxation was antagonized with i.v. 0.01 mg/ kg glycopyrrolate and 0.04 mg/kg neostigmine. Tracheal extubation was done after patients’ respond to the verbal command and adequate spontaneous ventilation. Patients were transferred to the post-anesthesia care unit (PACU) until they fulfill discharge criteria (moderate Aldrete score > 9) [12] and then moved to the ward.

### Intervention and randomization

Randomisation was performed before induction of anesthesia by an independent anesthesiologist responsible for patient allocation. Computer-generated randomization code (Random Allocation Software Version 1.0) with opaque envelopes containing sequential number was used for randomisation. Patients were allocated to one of the two groups. Patients in group A (control group without tapering of remifentanil) were maintained with 0.3 μg/kg/min remifentanil until the end of surgery. In group B (experimental group with tapering of remifetanil), remifentanil was stepwise tapered (0.3 μg/kg/min for 10 minutes → 0.2 μg/kg/min for 10 minutes → 0.1 μg/kg/min for at least 10 minutes) at least for 30 minutes before the end of surgery by the anesthesiologist who is in charge of the patients. Patients and outcome assessors were blinded to group assignment.

### Outcome measures

Patients’ characteristics including age, gender, weight, height, BMI, ASA physical class, total dose of remifentanil, and information on surgery were collected. The primary outcome was the postoperative pain. Patients were asked to evaluate their level of postoperative pain at 30 min, 2 h, 6 h, 12 h, and 24 h postoperatively using NRS by blinded outcome assessors.

Secondary outcomes were information on the rescue analgesic, the concentration of desflurane and awakening time (the time interval from remifentanil discontinuation to extubation). An i.v. bolus dose of ketorolac (30 mg) was administered as a rescue analgesic for the patient with pain scores > 30. The number of the rescue analgesic and the time interval from the discontinuation of the remifentanil to the first analgesic requirement was also observed. Episodes of postoperative nausea, vomiting and other adverse effects such as shivering, dizziness, headache, or drowsiness were recorded postoperatively.

### Statistics

In a preliminary study, pain scores in 10 patients without tapering of remifentanil were 50 (25) at postoperative 2 h. Reduction the mean pain scores of 20 by the titration of remifentanil was considered to be clinically important. Twenty six patients in each group (power of 80% and type I error of 5%) was calculated with the analysis using sample size software (PASS 2005®, NCSS, USA). Assuming a 20% dropout rate, the final sample size was determined to be 31 patients per group.

Test of normality and analysis of data were performed using SPSS version 15.0 for Windows (SPSS, Chicago, IL, USA). Mann–Whitney test was used to compare pain scores, the end-tidal concentration of desflurane, awakening time and the time to the first analgesic requirement between the two groups. *Chi*-*square* test or *Fisher* exact test was used to analyze categorical variables (the number of rescue analgesics and adverse events). The correlation between the end-tidal concentration of desflurane at the stop of remifentanil and awakening time was analyzed with Spearman’s rho test. Data are presented as median (range) or count (n). Two-sided P-values of < 0.05 were considered statistically significant.

## Results

**Figure 1:**
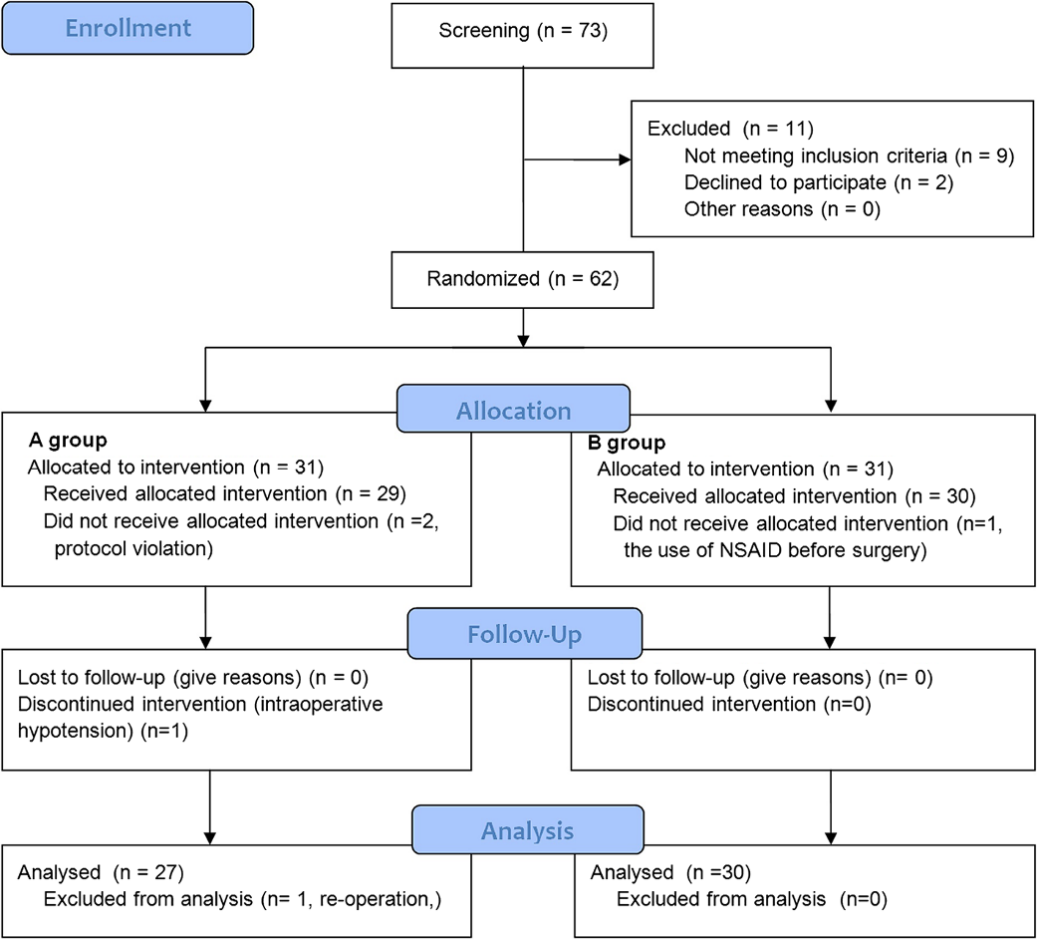
**Consort flow diagram.** Seventy three patients were assessed for eligibility and 11 patients were excluded because of exclusion criteria (9 patients) and patient refusals (2 patients). Sixty two patients were randomized for the study and 57 patients were included in the final analysis. Five patients (4 patients from group A and 1 patient from group B) were excluded during the study period for protocol violations, intraoperative hypotension, re-operation and the use of NSAID on the day of operation.

Seventy three patients were assessed for eligibility and 11 patients were excluded because of exclusion criteria (9 patients) and patient refusals (2 patients). Sixty two patients were randomized for the study and 57 patients were included in the final analysis. Five patients (4 patients from group A and 1 patient from group B) were excluded during the study period for protocol violations, intraoperative hypotension, re-operation and the use of NSAID on the day of operation (Figure 1). Patients and surgical characteristics are presented in the Table 1.

**Table 1:** **Patients and surgical characteristics**

	Treatment Groups	
Characteristic	A group (n= 27)	B group (n= 30)
Age (years)	44 (21-63)	42 (29-66)
Male/female (%)	14 (52)/13 (48)	15 (50)/15 (50)
Weight (kg)	66 (46-92)	69 (49-92)
Height (cm)	164 (145-180)	166 (148-183)
BMI	25(18-33)	25 (20-31)
ASA physical class (I/II)	19 (70)/8 (30)	23 (77)/7 (23)
Indication of thyroidectomy, n,(%)		
Thyroid cancer	25 (93)	28(93)
Thyroid nodule	2 (7)	2 (7)
Duration of operation (min)	83 (40-160)	79 (50-175)
Duration of anesthesia (min)	120 (75-155)	112 (70-200)
Remifentanil dose (mg)	1.8 (1.2-2.7)	1.7 (0.9-3.3)
Type of Operation		
Hemithyroidectomy	1 (4)	3 (10)
Total thyroidectomy	1 (4)	0 (0)
Hemithyroidectomy with neck dissection	6 (22)	4 (13)
Total thyroidectomy with neck dissection	19 (70)	23 (77)

Values are given as median (range) or number of patients (%). A group: no tapering group, B group: tapering group. BMI: body mass index.

Patients in group B reported significantly less pain than did those in group A 30 min (20 [0–80] *vs*. 50 [0–100], P = 0.002) and 2 h (30 [10–60] *vs*. 40 [20–80], P = 0.018) after surgery (Table 2). Additionally, rescue analgesics were less frequently required in group B than in group A (2 [1-3] *vs*. 3 [2,3], P = 0.039, Table 2). There is no statistically significance in the time intervals from the discontinuation of remifentanil to the administration of the first rescue analgesic between the two groups (P = 0.742, Table 2).

**Table 2:** **Postoperative pain scores, and rescue analgesics**

Treatment Groups			
Characteristic	A group (n = 27)	B group (n = 30)	P value
Postoperative pain scores			
30 min	50 (0–100)	20 (0–80)	0.002
2 h	40 (20–80)	30 (10–60)	0.018
6 h	30 (20–50)	30 (20–50)	0.966
12 h	27 (20–40)	26 (10–60)	0.593
24 h	20 (0–40)	18 (10–30)	0.330
Rescue analgesics, n	3 (2–3)	2 (1–3)	0.039
First use of rescue analgesics (min)	32 (10–72)	32 (5–417)	0.742

Values are given as median (range). A group: no tapering group, B group: tapering group; NRS: numerical rating scale (0 = no pain, 100 = the worst possible pain).

**Figure 2:**
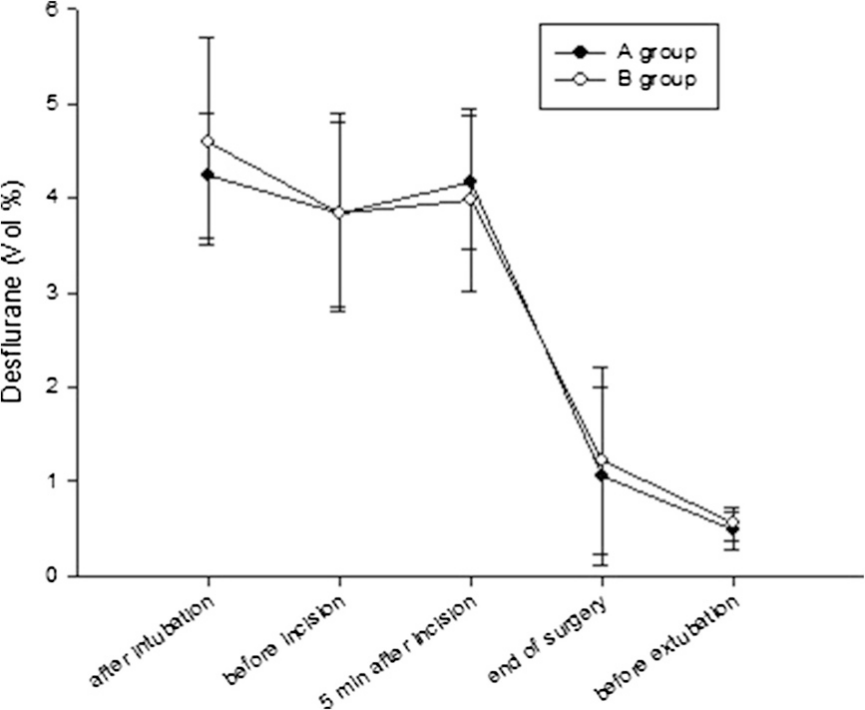
**End-tidal concentration of desflurane during the operation.** Values are given as mean (SD). A group: no tapering group, B group: tapering group.

No significant difference was observed in the end-tidal concentration of desflurane between the two groups (Figure 2). There was statistically significant correlation between the end-tidal concentration of desflurane at the stop of remifentanil and awake time (P = 0.002). No significant differences were observed in other adverse events including nausea, vomiting, shivering, headache, dizziness and drowsiness after surgery (Table 3).

**Table 3:** **Awakening time and postoperative adverse events**

	Treatment Groups		
	A group (n = 27)	B group (n = 30)	*P*value
Awakening time (min)	6 (1–15)	4 (2–14)	0.086
Nausea	7 (26)	6 (20)	0.594
Vomiting	1 (4)	1 (3)	1.000
Shivering	3 (11)	3 (10)	1.000
Headache	12 (44)	9 (30)	0.259
Dizziness	2 (7)	8 (27)	0.083
Drowsiness	1 (4)	0 (0)	0.474

Values are given as median (range) or number of patients (%). A group: no tapering group, B group: tapering group. Awakening time: the time interval from remifentanil discontinuation to extubation.

## Discussion

This study was designed to assess whether intraoperative tapering of remifentanil could diminish pain and rescue analgesics postoperatively. The results of this study showed that stepwise tapering of remifentanil at the end of the surgery reduced the postoperative pain scores and the requirement of rescue analgesics.

Patients in control group showed higher pain scores and required more rescue analgesics than those in tapering group did during immediate postoperative period. The possible explanation for this finding may be the development of opioid induced hyperalgesia or acute opioid tolerance. Opioid induced hyperalgesia is defined by the increased sensitivity by pain whereas acute opioid tolerance is a decreased sensitivity to opioid analgesics after administration of high dose opioids [6]. However, it is difficult to discriminate acute opioid tolerance and opioid induced hyperalgesia since a mixture of these phenomena may be associated with the infusion of high-dose opioids [13]. In addition, both phenomena are known to be induced by the activation of the NMDA receptor and the subsequent central sensitization of nociceptive system [8]. One of the current review of clinical data suggested that increased postoperative pain scores and subsequent greater requirement of analgesics may be associated with acute opioid tolerance, and the greater demand of opioids at a later recovery stage could be related with opioid induced hyperalgesia after high-dose remifentanil anesthesia [14].

Remifentanil differs from other opioids in that it directly acts on NMDA receptor [15]. Glycine, a vehicle of remifentanil, is bound with glutamate of the NMDA receptor during activation [16]. The present study showed clinically that stepwise tapering of remifentanil at the end of surgery reduced the postoperative pain.

Remifentanil is a short-acting opioid with a predictable rapid recovery [1]. In the current study, high-dose remifentanil (0.3 μg/kg/min) was used throughout the operation before intervention. Then, patients with control group were maintained with 0.3 μg/kg/min remifentanil until the end of surgery whereas patients with tapering group received titration of remifentanil (0.3 - > 0.2- > 0.1 μg/kg/min) for at least 30 minutes. Several previous investigation on opioid induced hyperalgesia infused high dose of remifentanil from 0.2 -0.4 μg/kg/min [2,3,17]. Guignard et al. [2], evaluated postoperative pain scores in patients after major abdominal surgery who had received high-dose remifentanil (mean dose 0.3 μg/kg/min) perioperatively and found that patients with high-dose remifentanil anesthesia needed morphine earlier and greater doses to achieve satisfactory analgesia postoperatively. In the study by Schmidt et al., patients undergoing eye surgery received either a high (0.4 μg/kg/min) or a low (0.1 μg/kg/min) dose of remifentanil and reported opioid-induced hyperalgesia [17]. Surgical site pain was assessed and postoperative values were compared with preoperative baseline measurements at other sites with cold and cold pressor test at 30 and 90 min after discontinuation of remifentanil [17]. Previous investigation of patients undergoing major abdominal surgery suggested hyperalgesia after high dose remifentanil and preventive effect of ketamine for opioid-induced hyperalgesia [3]. Patients with intraoperative high-dose remifentanil (0.4 μg/kg/min) showed larger areas of hyperalgesia surrounding the wound and the request of higher doses of postoperative opioid for pain control [3]. On the other hand, patients with low-dose remifentanil (0.05 μg/kg/min) alone, or higher-dose remifentanil and ketamine, reported similar areas of hyperalgesia and required similar doses of postoperative morphine [3]. In the study of comparing the effects of ketamine and paracetamol on preventing remifentanil induced hyperalgesia, anesthesia was maintained with 0.4 μg/kg/min remifentanil infusion in all groups and it was shown that ketamine and paracetamol were both effective in preventing remifentanil induced hyperalgesia [18].

Previous investigations with various anesthetics reported that anesthetics also affect opioid induced hyperalgesia since anesthetics have NMDA antanogist effect. Propofol suppresses NMDA receptor and there is a possibility of confusion for the interpretation of the results [19,20]. Therefore, balanced anesthesia with desflurane-high dose remifentanil was used for this study [21]. The concentration of desflurane was adjusted to maintain the spectral entropy between 40 and 60 and there was no difference in the end-tidal concentration of desflurane between the two groups.

A few limitations should be considered. The first limitation is the dose of remifentanil infusion during intervention. There was no established tapering regimen and the dose of remifentanil was stepwise reduced for at least 30 minutes (0.3 μg/kg/min for 10 minutes → 0.2 μg/kg/min for 10 minutes → 0.1 μg/kg/min for at least 10 minutes) based on the previous animal experiment [11]. Second, NSAID was used as a rescue analgesic instead of opioids in this study since patients with thyroidectomy have a high risk of postoperative nausea and vomiting. Therefore, it is insufficient to explain the result of this study as opioid induced hyperalgesia. Third, only postoperative pain score was assessed without direct measurement of somatosensory threshold after anesthesia. Postoperative surgical site pain should have been assessed and compared with preoperative value. In addition, postoperative surgical site pain should have been compared with those of other sites with cold and cold pressor test after discontinuation of remifentanil. Thyroidectomy was chosen in this study because balanced anesthesia with inhalation agent and remifentanil is used for the maintenance of anesthesia. During thyroidectomy, tracheal stimulation with endotracheal cuff pressure needs adequate depth of anesthesia with high dose of remifentanil. However, it is difficult to detect statistical difference between the two groups after postoperative 2 hr and this phenomenon seems to be related with lesser degree of pain after thyroidectomy (10–30 NRS). It remains to be seen whether increased pain scores could be associated with hyperalgesia and further study is needed to evaluate effect of tapering of remifentanil on the opioid induced hyperalgesia with direct measurement of somatosensory threshold before and after anesthesia.

## Conclusion

In conclusion, the results of this study suggested that stepwise tapering of remifentanil at the end of surgery could decrease postoperative pain and rescue analgesics after desflurane-high dose remifentanil anesthesia in patients with thyroidectomy. The further study on the exact mechanism of this phenomenon is needed.

## Abbreviations

NRS: Numerical rating scale
NMDA: N-methyl-D-aspartate
NSAIDs: Nonsteroidal anti-inflammatory drugs
COX: Cyclooxygenase
ASA: American Society of Anesthesiologist
PACU: Post-anesthesia care unit
